# Comparison of three-dimensional digital analyses and two-dimensional histomorphometric analyses of the bone-implant interface

**DOI:** 10.1371/journal.pone.0276269

**Published:** 2022-10-14

**Authors:** Jeong-Min Hong, Ung-Gyu Kim, In-Sung Luke Yeo

**Affiliations:** 1 Department of Prosthodontics, School of Dentistry, Seoul National University, Seoul, Korea; 2 Department of Prosthodontics, School of Dentistry and Dental Research Institute, Seoul National University, Seoul, Korea; Thamar University, Faculty of Dentistry, YEMEN

## Abstract

Histological analysis is considered to be the gold standard method of evaluating osseointegration around a bone-implant. However, this method requires invasive specimen preparation and is capable of representing only one plane. By comparison, micro-computed tomography (μCT) is a fast and convenient method that offers three-dimensional information but is hampered by problems related to resolution and artifacts, making it a supplementary method for osseointegration analysis. To verify the reliability of μCT for osseointegration evaluation, this animal model study compared bone-to-implant contact (BIC) ratios obtained by the gold standard histomorphometric method with those obtained by the μCT method, using a rabbit tibia implant model. A sandblasted, large-grit, acid-etched (SLA) implant and a machined surface implant were inserted into each tibia of two rabbits (giving eight implants in total). Bone-implant specimens were analyzed using μCT with a spiral scan technique (SkyScan 1275) and histological sections were prepared thereafter. Three-dimensional (3D) reconstructed μCT data and four two-dimensional (2D) μCT sections, including one section corresponding to the histologic section and three additional sections rotated 45°, 90°, and 135°, were used to calculate the BIC ratio. The Pearson’s test was used for correlation analysis at a significance level of 0.05. The histomorphometric BIC and the 2D-μCT BIC showed strong correlation (r = 0.762, P = 0.046), whereas the histomorphometric BIC and 3D-μCT BIC did not (r = -0.375, P = 0.385). However, the mean BIC value of three or four 2D-μCT sections showed a strong correlation with the 3D-μCT BIC (three sections: r = 0.781, P = 0.038; four sections: r = 0.804, P = 0.029). The results of this animal model study indicate that μCT can be used to complement the histomorphometric method in bone-implant interface analyses. With the limitations of this study, 3D-μCT analysis may even have a superior aspect in that it eliminates random variables that arise as a consequence of the selected cutting direction.

## Introduction

Osseointegration, which is essential for the successful clinical outcome of the dental implant that is assessed by criteria such as stability, function, and maintenance, is commonly evaluated by quantitative analyses of direct bone-to-implant contact (BIC) [1, 2]. Since the introduction of the concept of osseointegration by Brånemark in 1977, measurement of the BIC ratio on an undecalcified histological section using light microscopy has been regarded as the gold standard analysis method [3–6]. However, despite giving us much more qualitative information as well as quantitative ones, this histomorphometric approach is an inherently destructive and time-consuming method that requires intensive preparation processes such as sawing, grinding, and staining of the bone-implant section, all of which can result in technical errors. The invasiveness of the procedure also damages the specimen, precluding further examination, and does not allow evaluation of the specimen at various time points. Histomorphometric analyses also have the crucial drawback that only a small number of two-dimensional (2D) sections with the same orientation can be made; consequently, there is uncertainty over whether this method of measurement accurately represents the entire three-dimensional (3D) bone-to-implant surface. Therefore, despite the reliability of the histomorphometric method, a convenient and objective technique that allows 3D analysis of the BIC is needed.

Recently, micro-computed tomography (μCT) has emerged as a potential alternative method to assess the 3D morphology and architecture of BICs. This non-destructive and fast method offers not only information about the 3D structure, but can also be used to assess quantitative parameters such as bone density [7]. The drawback of μCT is that it has a lower resolution than light microscopy, causes the partial volume effect (PVE), and creates artifacts that can obstruct evaluation of the implant surface. To avoid such problems, a few groups have suggested analyzing the implant surface a few voxels away from the bone interface using μCT [8–12]. In addition, some studies have focused on identifying the optimum conditions for scanning, along with ways to minimize the occurrence of artifacts [13, 14]. Despite these efforts, the limitations of 3D-μCT have not been addressed fully and data generated using this method are currently only used to supplement conventional histomorphometric data [7].

Several studies have tried to verify the reliability of 3D-μCT data as a representation of the 2D histomorphometric data, but many still show conflicting results; moreover, the conditions for each study, such as the type of μCT device and analysis algorithm, were not standardized [10–12, 15, 16]. Accurate verification of the reliability of 3D-μCT data requires a number of criteria to be met: first, the 2D-μCT section corresponding to the histologic section must be defined exactly; second, optimized conditions for BIC analysis, such as segmentation threshold and region of interest (ROI), should be established by comparing the corresponding sections; and third, the BIC analysis of the reconstructed 3D-μCT data must be conducted under these conditions using an appropriate algorithm. To date, only a few studies have performed these three processes. One study suggested that three to four histologic sections, the maximum number that can be obtained along the longitudinal axis of one implant, are sufficient to represent the 3D osseointegration status [9]. However, only a few studies have considered the impact of various cutting directions on μCT results [17, 18].

The aim of this animal model study was to verify the suitability of the 3D-μCT BIC analysis method for osseointegration assessment by comparing it to the histologic BIC analysis method. Titanium implants with two different surfaces were implanted into the tibiae of two rabbits, and a spiral scanning technique, which is known to reduce artifacts associated with screw-shaped dental implants [19], was used to generate μCT images. Thereafter, histomorphometric BIC ratios were compared to the BIC ratios of the 2D-uCT sections that matched histologic sections, as well as the BIC ratios of reconstructed 3D uCT data. Additionally, the correlation between the BIC ratios of the 2D-μCT sections generated in variable cutting directions and reconstructed 3D uCT data was compared.

## Materials and methods

### Implants

Eight threaded titanium implants (Deep Implant Systems, Seongnam, Korea) were prepared for in vivo surgery; four were machined surface (turned) implants and four were sandblasted, large-grit, acid-etched (SLA) surface implants. The implants were 3.4 mm in diameter and 12 mm in length, and were made of grade 4 commercially pure titanium. A notch was made on the top of each fixture using a diamond bur to enable identification of an identical plane between the histomorphometric slide and μCT scan data.

### In vivo surgery

Eight implants were inserted in the tibiae of two normal male New Zealand White rabbits, which were aged 3 to 4 months, weighed 2.5 to 3 kg, and showed no signs of disease. The animal study protocol was approved by the Ethics Committee of the Animal Experimentation of the Institutional Animal Care and Use Committee (CRONEXIACUC 202103007; Cronex, Hwasung, Republic of Korea) and was performed in accordance with the Animal Research: Reporting of In Vivo Experiments (ARRIVE) guidelines [20].

**Fig 1:**
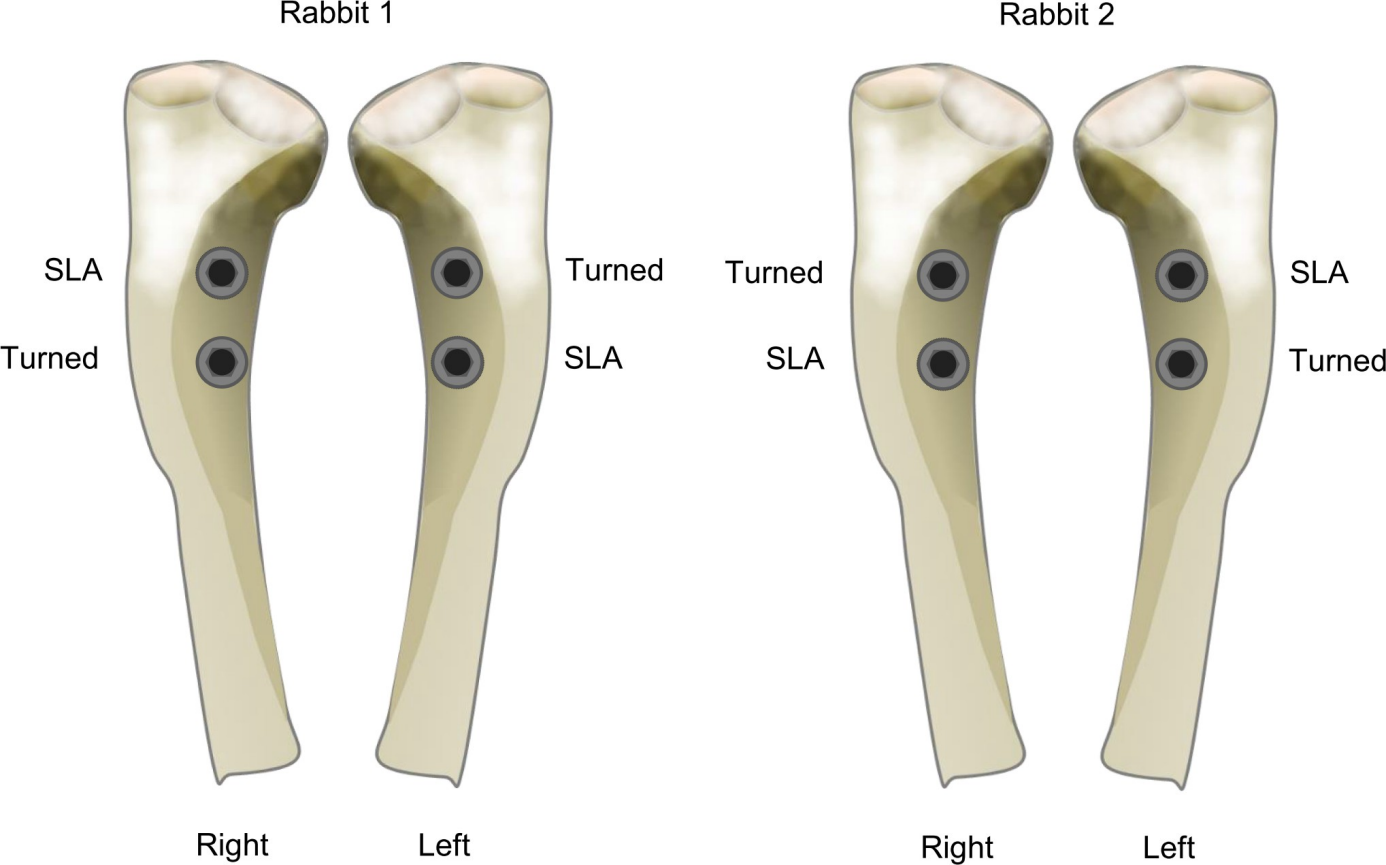
In vivo study design. Schematic illustration showing placement of the implants in the rabbit tibia model, considering complete randomization. SLA, sandblasted, large-grit, acid-etched implant.

The rabbits were anesthetized via intramuscular injection of tiletamine/zolazepam (15 mg/kg, Zoletil® 50, Virbac Korea Co. Ltd., Seoul, Korea) and xylazine (5 mg/kg, Rompun™, Bayer Korea Ltd., Seoul, Korea). Before surgery, the skin on the surgical site was shaved and disinfected with betadine, and then the rabbits were given an intramuscular administration of the antibiotic cephalosporin (Cefazolin; Yuhan Co., Seoul, Korea). Each tibia was then locally injected with 0.9 mL of 2% lidocaine with 1:100,000 epinephrine (2% Lidocaine HCL Injection, Huons Co., Ltd, Seongnam, Korea). For implant placement, muscle dissection and periosteal elevation were performed after skin incision to expose the flat surfaces of the tibiae. Drilling was performed mono-cortically under saline irrigation with final diameters of 3 mm, according to the implant manufacturer’s protocol. After bone preparation, two implants were placed in each tibia, resulting in four implants per rabbit. Each implant was placed to make that the marked notch perpendicular to the long axis of the tibia [21]. SLA and turned surface implants were arranged according to a 2 × 2 Latin square for complete randomization with minimal sample size (Fig 1). Healing abutments were screwed in after implant placement and the muscle and periosteum were sutured with resorbable 4–0 Vicryl (Ethicon, Somerville, NJ, USA), while the skin was closed using 4–0 blue nylon (Ailee, Busan, Republic of Korea). Enrofloxacin (Komibiotril, Komipharm International, Siheung, Republic of Korea) was administered intramuscularly as an antibiotic for 3 days postoperatively.

Each rabbit was housed separately for 28 days and then sacrificed via an intravenous overdose of potassium chloride under anesthesia. After removal of the soft tissue, the implants were retrieved en bloc with adjacent bone, and were fixed in 10% neutral formaldehyde immediately.

### Micro-CT scanning

The implant-bone blocks were placed in a 50 mL Falcon conical tube (Fisher Scientific International, Hampton, NH) in a way that the long axis of the implant is perpendicular to the scanning beam. A SkyScan 1275 μCT scanner (Bruker, Kontich, Belgium) was used to perform a quantitative analysis of the surrounding bones. The scan time was 2 hours and 20 minutes, with an isotropic voxel size of 20 μm (resolution) and an acceleration voltage of 100 kV at 100 μA with a Cu filter (1 mm). A spiral scanning technique was used to reduce cone-beam artifacts common to round scanning [19]. For all samples, the exposure time was 217 ms with 0.1° of rotation step and frame averaging value was 4 with 0.003 mm of linear step. After scanning, the data were reconstructed using NRecon software (v.1.7.3.2; Bruker microCT, Kontich, Belgium) with a ring artifact correction value of 3 and 40% of beam hardening correction. All scans were reconstructed with the same contrast limit for the attenuation coefficient values (0 to 0.025). the contrast limit for the attenuation coefficient histogram is the most important parameter Subsequently, the reconstructed μCT data were aligned with the long axis of the implant using DataViewer software (v.1.5.4.0; Bruker microCT, Kontich, Belgium).

### Histomorphometry

After μCT scanning, undecalcified ground sections of bone-implant blocks were processed. The specimens were dehydrated with ethanol, embedded in light curing resin (Technovit 7200 resin, Heraeus Kulzer, Hanau, Germany), and then bisected longitudinally, along the plane, to include the notch and center of the healing abutment. One central section was prepared for each implant, resulting eight histological sections in total. Subsequently, the sections were ground to approximate thickness less than 50 μm and stained with hematoxylin and eosin. For histomorphometric analysis, images were obtained via light microscopy (BX51, Olympus, Tokyo, Japan) and the image analysis was performed using the ImageJ software (National Institutes of Health, Bethesda, MD, USA). The histomorphometric BIC ratio, defined as the total bone-to-implant contact length/geometrical length of implant surface, was calculated using the ‘measure’ tool of ImageJ at 40× magnification. All the BIC analyses were carried out by one blinded examiner.

### Micro-CT analysis

**Fig 2:**
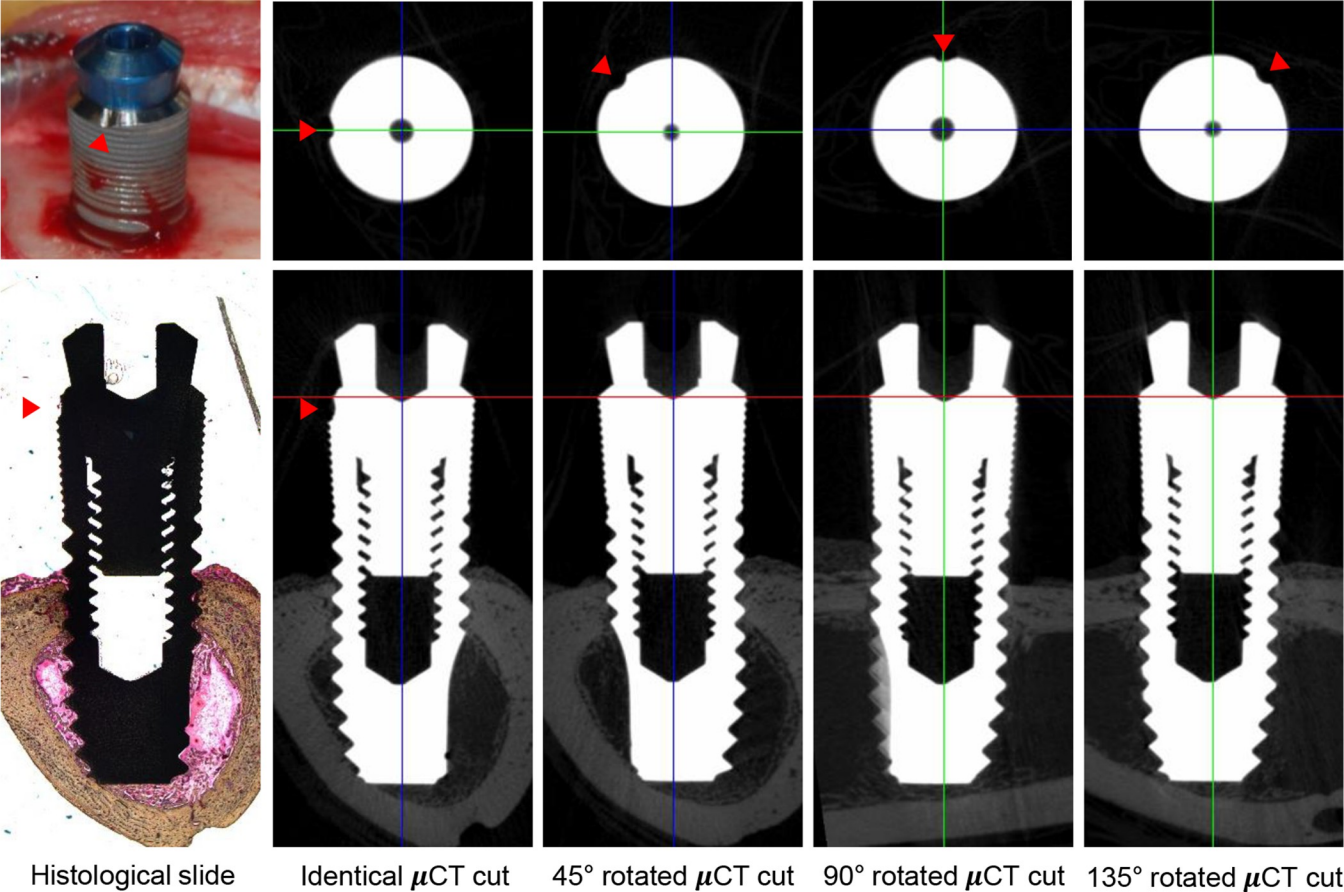
Two-dimensional micro-computed tomography (μCT) analysis of bone-implant sections. Representative 2D-μCT images of the histological-identical section of an implant and 45°, 90°, and 135° rotations of the plane. The red arrowheads indicate the position of the marker notch on the implant.

The 2D-μCT section that was identical to the histologic section was identified using DataViewer and CTAn software (v.1.18.4.0; Bruker microCT, Kontich, Belgium). After aligning the reconstructed μCT image to the plane that included the center of the implant and the marked notch, the matching slice to the histologic section was selected along the longitudinal view of the implant. Three additional sections that included the long axis of the implants were also obtained; these sections were rotated 45°, 90°, and 135° relative to the histological-identical section (Fig 2, S1 Fig).

**Fig 3:**
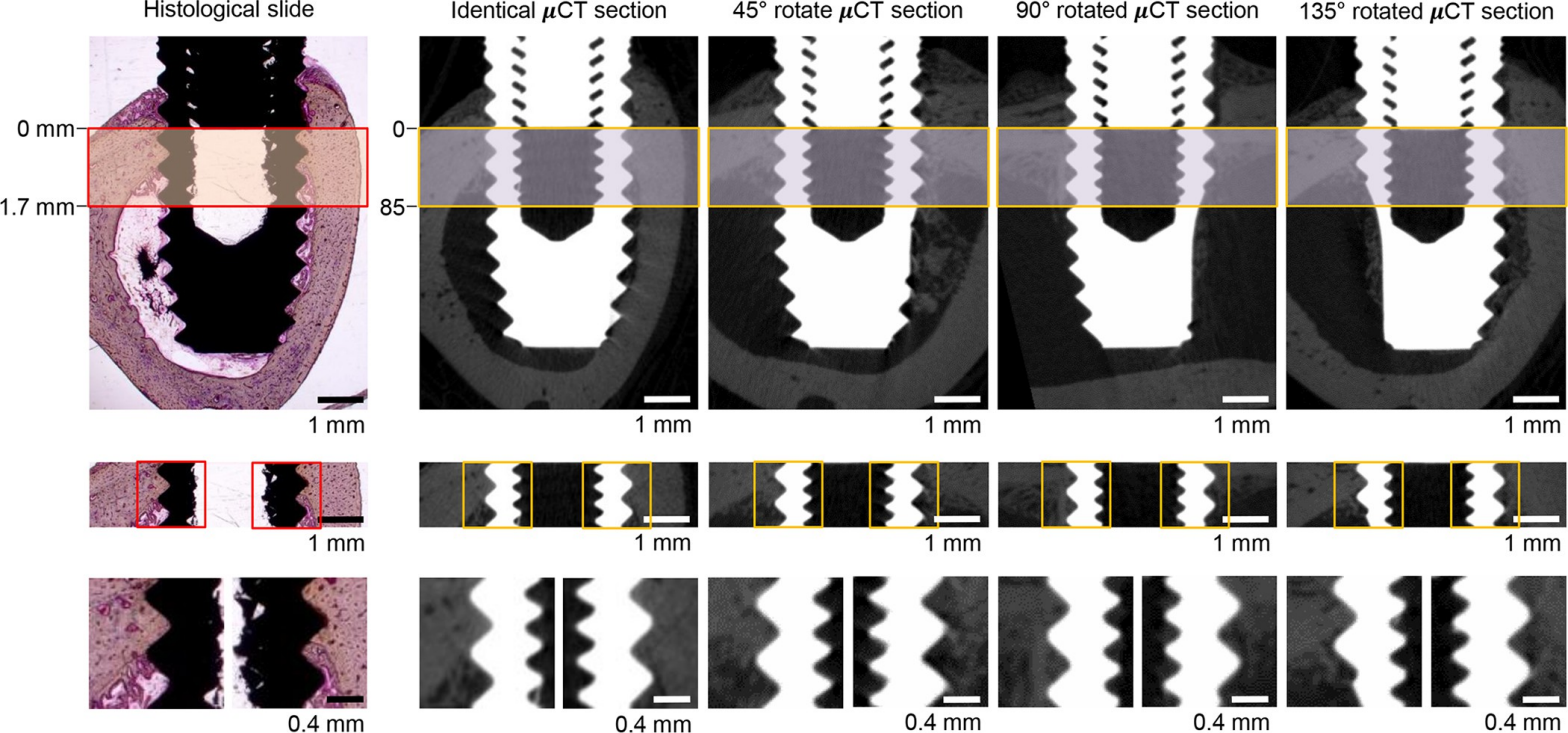
Region of interest for bone-to-implant contact ratio measurement. The boxed areas in the upper and middle panels show the cropped regions used for BIC ratio measurements. The analysis included 85 2D-μCT slices and a 1.7 mm histologic section from the bottom of the healing abutment. Each side of the implant surface was evaluated independently (lower panels).

**Fig 4:**
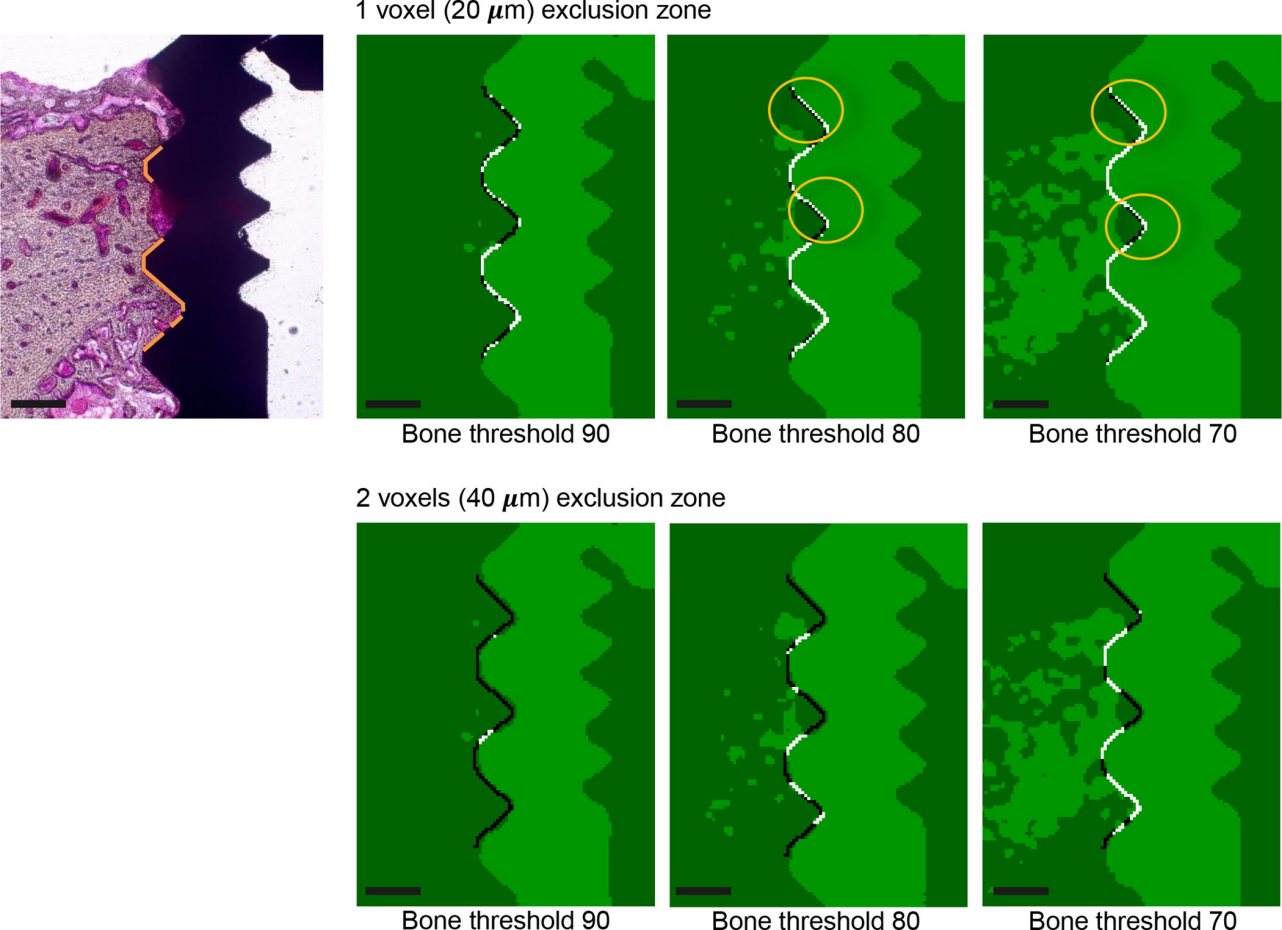
Relationship between the partial volume effect (PVE) and bone threshold at different region of interests. The orange lines on the histological section (left panel) represent actual bone-to implant-contact. The region of interest (ROI) is represented by a black line in the binarized 2D-μCT sections, and the white area in the ROI indicates the presence of bone under the given bone threshold. A ROI located one voxel (20 μm) away from the implant surface (upper panels) resulted in false positive bone-to-implant contact (orange circles). A ROI located two voxels (40 μm) away from the implant surface (lower panels) resulted in no false positive bone-to-implant contact caused by the PVE. Scale bars = 0.5 mm.

BIC assessment was performed within a 1.7 mm area along the long axis of the implant (crestal portion), beginning from the bottom of the healing abutment, such that 85 slices in the μCT data were cropped (Fig 3). To measure the 2D-μCT BIC ratio, the ROI was set between the second and third voxel from the surface of the implant, to avoid titanium-induced artifacts. Such artifacts typically occur 20 to 40 μm from the implant surface and were not completely avoided when the ROI was set to one voxel away from the implant surface (Fig 4). Thereafter, the implant threshold and bone threshold were determined manually by the best visual agreement using identical 2D slices. The same thresholds were applied to all samples, and each side of the implant was analyzed independently in the 2D analysis. Finally, bone and implants were binarized with each threshold. BIC assessment was performed on the four different 2D-μCT sections (Fig 3) and 3D-μCT reconstructed data, using the ROI and threshold specified above. All the BIC analyses were carried out by one blinded examiner.

### Statistical analysis

Independent t-tests were used to compare the BIC ratios of the two different implant surfaces determined using 2D histologic sections and 2D-μCT and 3D-μCT data. Pearson’s correlation coefficients were used to evaluate correlations between the BIC ratios determined using 2D histologic sections and those generated using 2D-μCT or 3D-μCT data. In addition, correlations between the 3D-μCT BIC ratios and the mean 2D-μCT BIC ratios of sections cut in different directions were also examined. All statistical analyses were performed with R software (v.4.1.0; R Foundation for Statistical Computing, Vienna, Austria) and P < 0.05 was considered to be statistically significant.

## Results

### In vivo surgery

A total of eight titanium implants were inserted in the tibiae of two male rabbits (one turned surface implant and one SLA surface implant per tibia per rabbit). Routine clinical inspections showed that healing progressed uneventfully after surgery and there were no clinical signs of infection at the time of sacrifice. Because all samples showed successful osseointegration, no samples were excluded in the BIC analysis.

### Histomorphometric analysis

Bone-implant blocks were prepared 4-weeks post-surgery and, after μCT scanning, were processed for histomorphometric analyses. The overall mean BIC determined using histological sections of the bone-implant blocks was 42.4% [standard deviation (SD) 14.4; range 25.6–72.7]. The mean BIC of the SLA surface implants was 50.5% [SD 16.0; range 34.6–72.7], whereas that of the turned surface implants was 34.3% [SD 7.3; range 25.6–42.5] (Table 1). The difference between the BIC ratios of the SLA and turned surface implants determined using histological sections was not statistically significant (P = 0.116).

**Table 1:** Bone-to-implant contact (BIC) ratios of the implants determined using histologic sections, 2D-μCT, and 3D-μCT images, and the correlations between the different methods.

	Histo BIC	2D-μCT BIC	3D-μCT BIC	Correlation^a^	Correlation^a^
	(mean ± SD)	(mean ± SD)	(mean ± SD)	(Histo / 2D-μCT)	(Histo / 3D-μCT)
Total	42.4 ± 14.4	38.7 ± 12.1	52.1 ± 5.9	0.762* (P = 0.046)	-0.375 (P = 0.385)
SLA^b^	50.5 ± 16.0	38.1 ± 15.7	48.6 ± 5.1		
Turned	34.3 ± 7.3	39.4 ± 9.6	55.7 ± 4.8		

^a^ Pearson’s correlation coefficient.

^b^ Sandblasted, large-grit, acid-etched implant.

*Statistically significant.

### Micro-CT analysis

As mentioned above, a distance of two voxels (40 μm) from the maximum titanium absorption values was found to avoid the PVE and was therefore optimal for the 2D-μCT analysis (Fig 4). The threshold gray-level for the bone was 70 (bone mineral density: 686 mg/cm^3^ HA), whereas that for the titanium implant was 170 (bone mineral density: 1667 mg/cm^3^ HA) on an 8-bit scale (0–255). The threshold levels were related to bone mineral density, using a calibration phantom [22].

Table 1 shows the mean BIC ratios calculated using the histologically matching 2D-μCT sections and the reconstructed 3D-μCT images. For each method, the difference between the BIC ratios of the SLA and turned surface implants was not statistically significant (2D-μCT, P = 0.887; 3D-μCT, P = 0.0874).

### Correlations between the BIC ratios determined using histologic, 2D-μCT, and 3D-μCT images

A Pearson’s correlation analysis revealed a significant correlation between the BIC ratios calculated using the histological sections and identically matched 2D-μCT images (P = 0.046); however, there was no significant correlation between the BIC ratios calculated using the histomorphometry and 3D-μCT images (P = 0.385) (Table 1, S2 Fig).

Next, BIC ratios were determined using three other 2D-μCT sections that were rotated 45°, 90°, and 135° relative to the histological-matched 2D-μCT section. There was no correlation between the BIC ratios determined using the 3D-μCT image and the mean value of two 2D-μCT sections (identical section and section rotated 90°); however, there was a strong correlation between the BIC ratio determined using the 3D-μCT image and the mean value determined using all four 2D-μCT sections (identical section and sections rotated 45°, 90°, and 135°) (Table 2, S3 Fig).

**Table 2:** BIC ratios determined using the indicated numbers of 2D-μCT sections, and the correlations between them and the BIC ratio determined using 3D-μCT images.

	2D-μCT BIC	2D-μCT BIC	2D-μCT BIC	2D-μCT BIC	3D-μCT BIC
	1 section^a^	2 sections^b^	3 sections^c^	4 sections^d^	
	(mean ± SD)	(mean ± SD)	(mean ± SD)	(mean ± SD)	(mean ± SD)
Mean BIC	38.7 ± 12.1	39.1 ± 11.5	35.8 ± 9.1	35.5 ± 8.3	52.1 ± 5.9
Correlation^e^	0.477 (P = 0.279)	0.628 (P = 0.131)	0.781* (P = 0.038)	0.804* (P = 0.029)	

^a^ Histological-identical section.

^b^ Histological-identical section and 90° rotated section.

^c^ Histological-identical section and 45° and 90° rotated sections.

^d^ Histological-identical section and 45°, 90°, and 135° rotated sections.

^e^ Pearson’s correlation coefficient (between the BIC ratio determined using the indicated number of 2D sections and that determined using the 3D image).

*Statistically significant.

## Discussion

In this animal model study, we compared the BIC ratio calculated using a 2D histological method with those calculated using an identically matched 2D-μCT section and a 3D-μCT image. Despite the advantages of μCT-based implant surface analysis, including multifaceted and chronological analyses through 3D reconstruction and μCT scanning at various time points, it is currently only used as a supplementary method due to issues regarding resolution and artifacts. Most commercial implants have threaded geometry, which generates more artifact-related problems than simple geometry titanium. Therefore, spiral scanning, which reduces the occurrence of artifacts generated by threaded-type implants [19], was used in this study to obtain μCT data. In terms of chronological evaluation, there is much to be improved even in the in vivo situation of small animals, such as scan time, fixation of the specimen, radiation dose, and field of view. At the present, further studies are required to utilize μCT for clinical investigation, especially on titanium implants [8, 12, 23]. In the case of the PVE, which occurs due to resolution problems that arise when two substances with different attenuation coefficients are in contact, the higher the resolution, the lower the impact [10, 14]. However, despite the improved resolution of μCT, it is still lower than that of light microscopy; thus this study required the use of an exclusion zone of 40 μm to eliminate the PVE completely, as suggested in previous studies (Fig 4) [8–12]. Although the exclusion zone of 40 μm used in this study is very small and may reflect the bone contact of the implant surface, it deviates from the original definition of osseointegration, i.e., direct bone-to-implant contact that ensures the fixation of a clinically established implant. To improve limitations due to the discrepancy of the ROI, an optimal method to improve resolution and eliminate artifacts needs to be established. In the case of the PVE, which occurs due to resolution problems that arise when two substances with different attenuation coefficients are in contact, the higher the resolution, the lower the impact [10, 14]. However, despite the improved resolution of μCT, it is still lower than that of light microscopy; thus this study required the use of an exclusion zone of 40 μm to eliminate the PVE completely, as suggested in previous studies (Fig 4) [8–12]. Although the exclusion zone of 40 μm used in this study is very small and the measurement may reflect the bone contact of the implant surface, it deviates from the original definition of osseointegration which ensures the fixation of a clinically established implant. To improve limitations due to the discrepancy of the ROI, an optimal method to improve resolution and eliminate artifacts needs to be established. Previous studies reported approximately 50 μm of uncertainty in μCT-based implant surface analysis utilizing the reference values obtained with histomorphometry, which was quantitatively unstandardized [14, 16, 24]. Due to the factors influencing the accuracy of μCT scanning and μCT-based analysis, further quantitative studies to standardize correction of the systematic errors and to use regression equations are needed for the true-BIC value [14, 24].

We performed a step-by-step procedure to verify the μCT-based BIC analysis. Identical μCT sections that matched the histologic sections were identified using a marked notch on the top of the implant as a reference point. Subsequently, the threshold that corresponded most to the bone-to-implant contact pattern of the histologic section was derived. The same threshold was then applied to the 3D-μCT analysis. Although the BIC ratios determined using the 2D-μCT analysis correlated with those determined via histologic analysis, a similar correlation was not seen between the 3D-μCT-determined and histological-determined BIC ratios. This finding suggests that the histologic section, which is limited to two dimensions, may not provide an accurate representation of the 3D in vivo condition. A previous study demonstrated that three to four histologic sections per implant can properly represent the whole 3D situation without bias caused by selection of a single cutting direction [9]. However, obtaining three to four sections per implant is a technically complex and, due to the cylindrical and tapered shape of the implant, it can be difficult to obtain a section that contains full lengths and diameters. Furthermore, the axis of the cross-section can be changed during the grinding process. Unlike the previous study [9], which used cross-sections cut in the same direction, our current animal model study analyzed 2D-μCT sections cut in different directions over the longitudinal axis of the implant. However, similar to previous results, even with multiple cutting directions, three to four sections were required for correlation between the BIC ratios of 2D-μCT sections and 3D-μCT images. The SD of the 3D-μCT BIC ratios was smaller than those of the histologic and 2D-μCT BICs (Table 1). This finding is probably attributable to the fact that 3D-μCT can eliminate variability arising from the random selection of 2D sections, which is a prominent limitation of the histologic method. The SD of the 2D-μCT BIC ratio also decreased as the number of sections (at different cutting directions) increased (Table 2), likely due to a reduction in intra-sample variability.

In the histological analysis, the BIC ratio of the SLA surface implant was higher than that of the turned surface implant, although the difference was not statistically significant (Table 1). The BIC ratios of the SLA and turned surface implants also did not differ significantly in the 2D-μCT and 3D-μCT analyses. This result may be due to the four-week healing period prior to BIC analyses, which may have allowed sufficient bone remodeling for both specimens, as seen in a previous study [25].

The selection process of bone and implant threshold is an important factor that affects the results of μCT analyses. The ROI is determined according of the titanium threshold of the implant and the analysis results can differ depending on the bone threshold within the ROI. The bone threshold can vary depending on the conditions of the experiment or individual differences between samples. Although this study used a small sample size to minimize the sacrifice of experimental animals, this remains as a limitation due to the fact that a large sample size would reduce individual differences and result in a more accurate verification. Even within the same sample, a smaller threshold can be obtained depending on the distance from the implant surface, due to the decrease of metal artifacts [12]. To utilize the digital aspect of μCT and develop an automated analysis method, further quantitative studies of bone thresholds are required [26, 27]. Also, the method of this ex vivo animal model study using μCT is not yet widely applicable for an in vivo or clinical setting due to problems such as long scan time, mechanical fixation of specimen, and radiation dosage [12, 23]. In the future, through further research on refinement and standardization of the technique, chronological in vivo scanning without sacrifice of experimental animals and clinical application seem to be feasible [28].

In addition to the limitations of small sample size and μCT, there was one more issue to be discussed in this study. Finding the 3D-μCT data that correspond to a specific histologic section, which is required to verify the μCT method, can also have a marked effect on the results. However, this process is not straightforward, due to errors that occur during processing of the histologic samples. In our current study, we identified samples with slight tilting of the longitudinal axis of the implant, caused by technical errors during specimen preparation. While it is possible to observe all sections freely using reconstructed μCT images, finding a matching plane by tilting the axis manually is very inefficient. Recently, some studies have explored the use of automatic registration of the 3D-μCT data to the 2D section [7, 28]. These studies, combined with the additional analyses of quantitative methods to determine thresholds mentioned previously, will ultimately aid the development of an improved 3D-μCT analysis method.

## Conclusion

With the limitations of this animal model study, 3D-μCT can be used for bone and implant interface analysis to complement the histomorphometric method. 3D-μCT analysis may even be superior to histomorphometry in that it enables observation of the entire implant and bone morphology and eliminates random variables caused by selection of the cutting direction.

## Supporting information

S1 Data Raw data. (XLSX)

S1 Fig 2D slices of all specimens. (TIF)

S2 Fig Correlation of the BIC ratio between histologic section and 2D-μCT or 3D-μCT. Scatterplots with line of best fit. (a) correlation between histomorphometry and 2D-μCT. (b) correlation between histomorphometry and 3D-μCT.
(TIF)

S3 Fig Correlation of the BIC ratio between 3D-μCT and the means of the 2D-sections cut in different directions. Scatterplots with line of best fit. Correlation between BIC ratio of the 3D-μCT and means of the different number of 2D sections cut in different directions. (a) 1 section, (b) 2 sections, (c) 3 sections, and (d) 4 sections.
(TIF)
