# Supplementary figures and images for: Comparison of three-dimensional digital analyses and two-dimensional histomorphometric analyses of the bone-implant interface
Source: PLoS One. 2022 Oct 14;17(10):e0276269. doi: 10.1371/journal.pone.0276269 (PMC9565376; doi:10.1371/journal.pone.0276269)

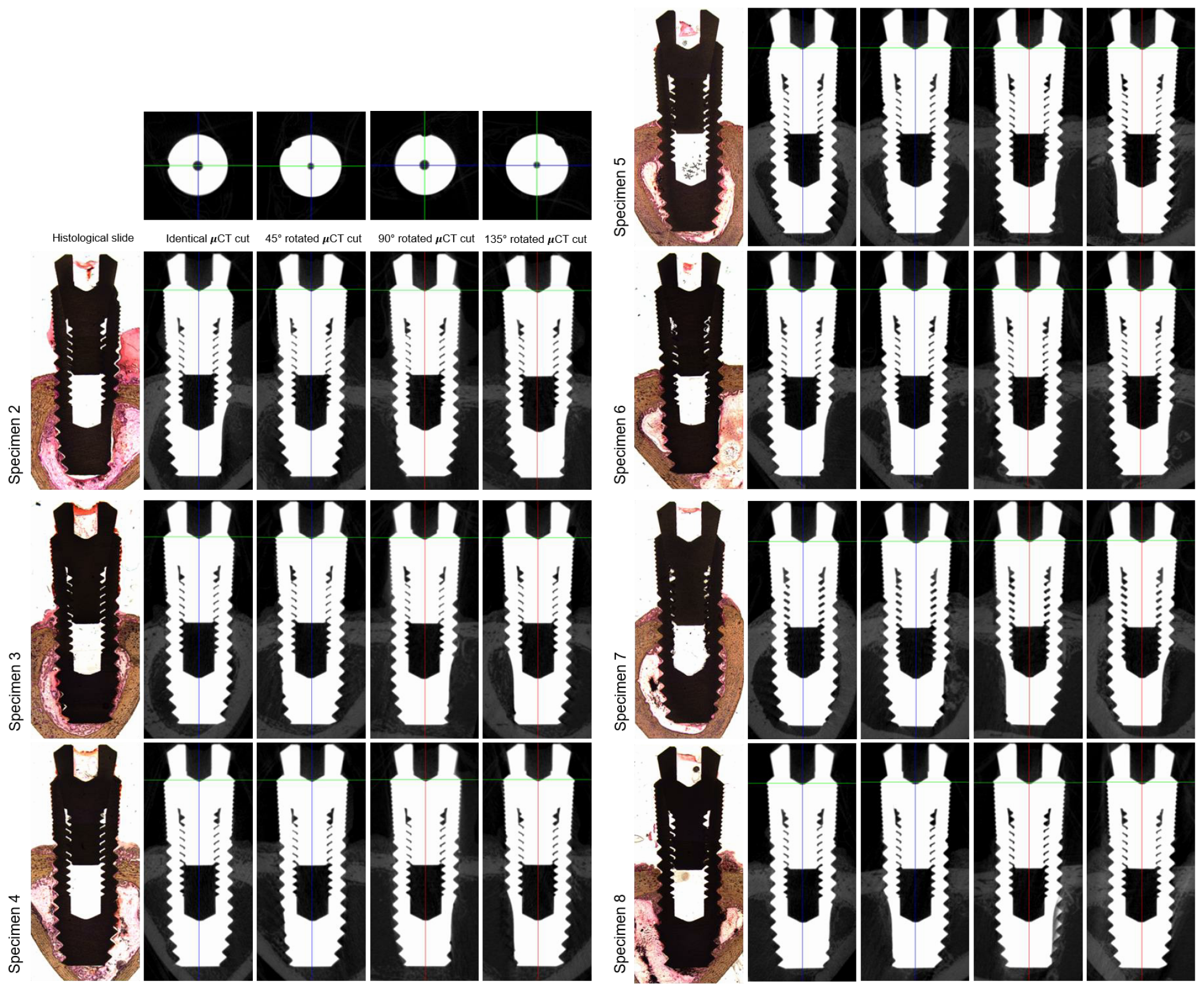

Supplement: S1 Fig — (TIF) [file pone.0276269.s002.tif]

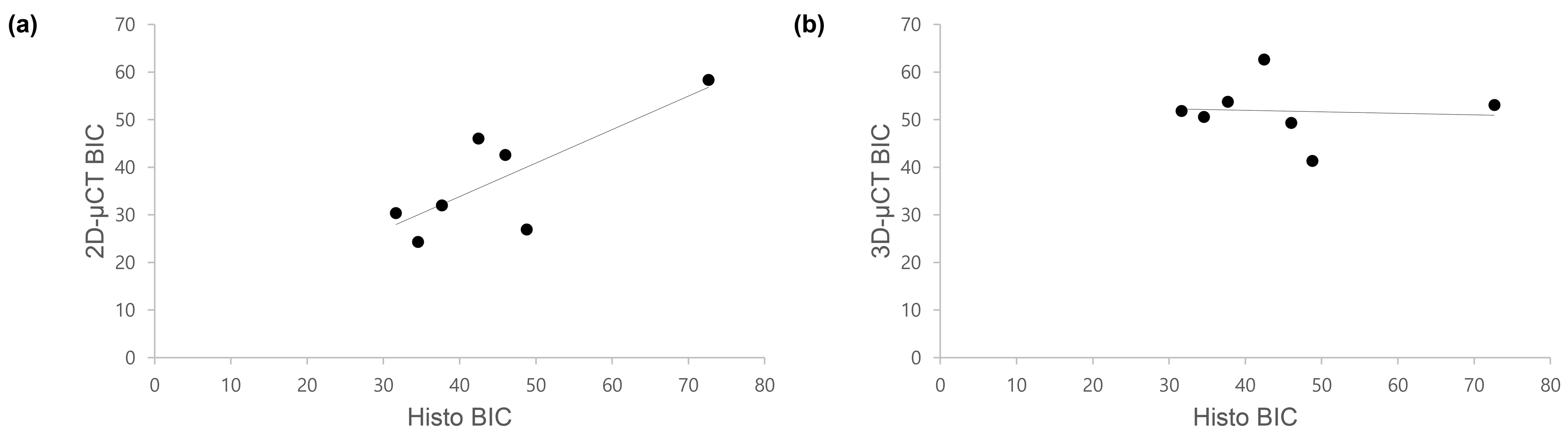

Supplement: S2 Fig — Scatterplots with line of best fit. (a) correlation between histomorphometry and 2D-μCT. (b) correlation between histomorphometry and 3D-μCT. (TIF) [file pone.0276269.s003.tif]

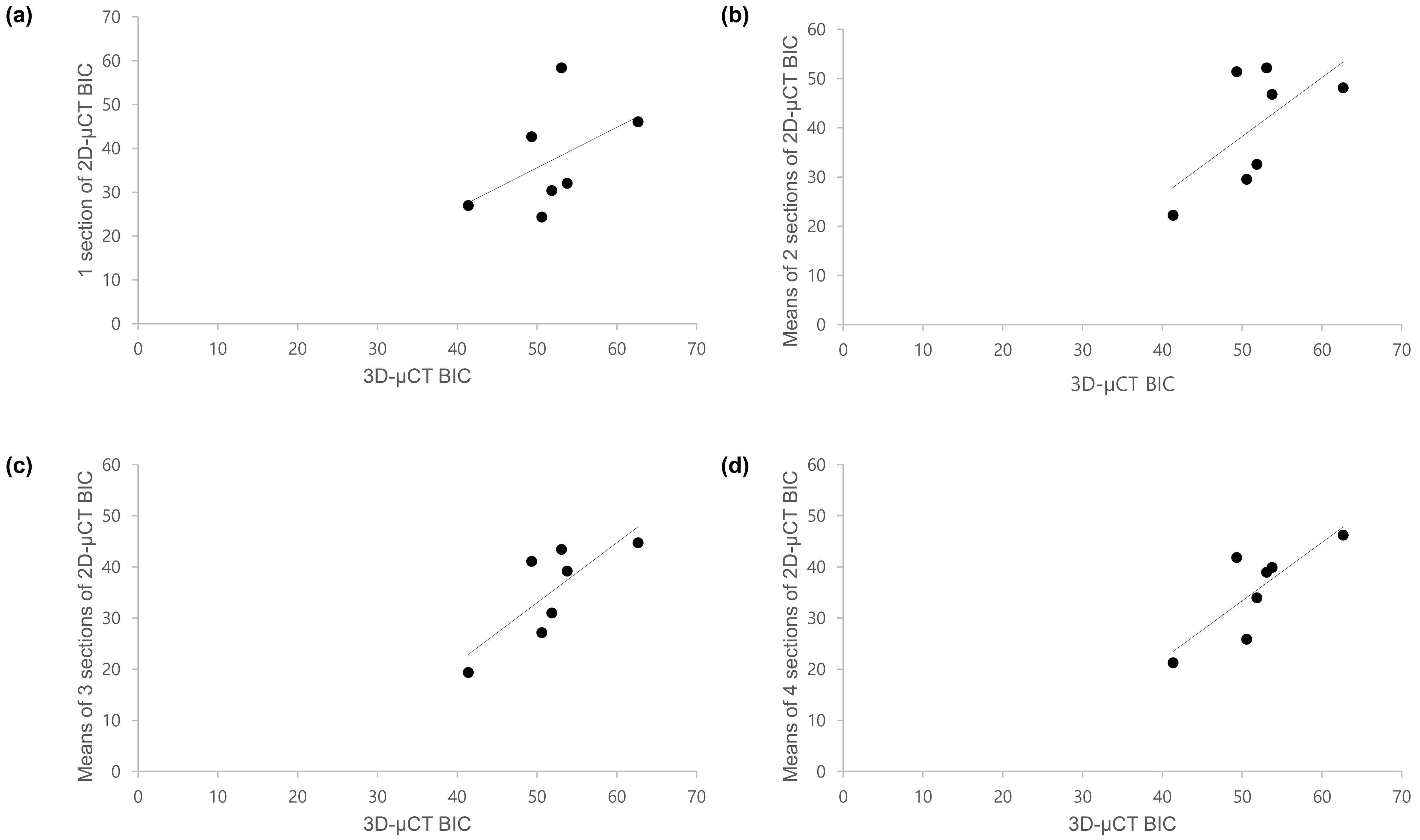

Supplement: S3 Fig — Scatterplots with line of best fit. Correlation between BIC ratio of the 3D-μCT and means of the different number of 2D sections cut in different directions. (a) 1 section, (b) 2 sections, (c) 3 sections, and (d) 4 sections. (TIF) [file pone.0276269.s004.tif]
